# Temporal decision making: it is all about context

**DOI:** 10.3758/s13420-022-00568-8

**Published:** 2022-12-29

**Authors:** Cemre Baykan, Zhuanghua Shi

**Affiliations:** Department of Experimental Psychology, Ludwig Maximilian University of Munich, Munich, DE Germany

## Abstract

Is there sufficient evidence to make a decision, or has enough time passed to justify making a decision? According to Ofir and Landau (2022, *Current Biology: CB*, *32*[18], 4093–4100.e6), these two questions are closely related: brain activity measured by EEG at the offset of stimulus presentation predicts the behavioral temporal decision, being influenced by the current context, and reflecting the relative distance to a decision threshold which is also context dependent.

During a lunch break, you open a cup of instant noodles, pour hot water over the noodles, cover, and wait. Time to open depends on your desired noodle softness and the sense of passage of time. Too early, noodles not ready. Too late, noodles too soft. Using a classic temporal bisection task, Ofir and Landau (2022) studied this type of time-based decision to understand the neurophysiological basis of interval timing and how it relates to the cumulative decision process.

In a temporal bisection task, participants are first presented with two references: the shortest and the longest durations. After familiarization, they are asked to classify durations that fall between the two references as “short” or “long.” Ofir and Landau (2022) measured electroencephalography (EEG) brain activity while participants performed this task. Similar to previous studies (for a review, see O’Connell & Kelly, 2021), they found a decision-related large positive deflection in a group of frontocentral electrodes at 300 to 500 ms after stimulus offset, known as the offset P300 or P3b. As stimulus duration increased, the amplitude of the offset P3b decreased linearly when the duration was close to the “short” reference, but leveled off when the duration was close to the “long” reference. Ofir and Landau (2022) interpreted this offset amplitude as the distance to the decision boundary in a temporal accumulation-to-bound model (Balcı & Simen, 2014). A bisection decision of “short” can be made when the presentation finishes before a decision threshold—the mean duration between the “short” and “long” references. No further trigger for the “long” decision is needed when the presentation time passes the mean duration. Linking the P3b amplitude to the distance to the decision boundary, Ofir and Landau (2022) could predict behavioral bisection performance accurately.

Interestingly, the pattern was similar for the short- and long-range durations. In their Experiment 3, participants performed the bisection task in two separate blocks: one with the subsecond (0.2–0.8 s) range and the other suprasecond (1–2 s) range. Short durations in both blocks, albeit differently in their absolute magnitude, elicited a similar large amplitude of the stimulus offset P3b (e.g., 0.2 s in the short block, and 1 s in the long block). This suggests the activation of the offset P3 does not reflect an absolute accumulation, but is rather context dependent, relative to the decision threshold set in that block. It is well-established that time estimation is susceptible to various background contexts, such as stimulus spacing and ensemble statistics (Zhu et al., 2021). Even with the same shortest and longest references, the mean duration of the two references is perceived as longer when more short durations are probed. But it is perceived as shorter when more long durations are sampled. The temporal bisection decision does not merely compare to the short and long references. Rather, the decision process considers the ensemble mean of the sampled durations as the decision boundary. The findings of Ofir and Landau (2022), consistent with the temporal context modulation, showed that the amplitude of offset P3b was determined by the relative distance to the ensemble mean of the probed range—a critical decision threshold. For example, as the longest duration in the short-range block, the 0.8-s target interval already passed the decision threshold, eliciting a low P3b amplitude. In contrast, larger than the 0.8-s interval in the short context, the shortest 1-s target interval induced the highest activation of the P3b in the long-range block. These results demonstrated that the offset EEG activities, along with the behavioral responses, were context dependent.

Contexts set our expectations. For example, we expect to wait a short amount of time for a traffic light to turn green than for a bus to arrive. These expectations influence our decision-making processes, as shown by Ofir and Landau (2022). Five minutes would be unexpected when waiting for a traffic light signal, but perfectly normal when waiting for a bus. In the temporal bisection task, a short stimulus duration would be more surprising than a long stimulus duration, because the decision process is likely already completed when the duration is longer than the average. Earlier research has found that the latency of the offset positivity components, such as the P300 or P3b, is positively correlated with participants’ reaction times (O’Connell & Kelly, 2021). A higher level of offset activity leads to a longer response time. The reaction time findings, along with the characteristic build-up of the P300, suggest that P300 activity may reflect the intensity of decision-making process (O’Connell & Kelly, 2021), which could also be interpreted as a surprise response to the stimulus offset, as Ofir and Landau (2022) noted. Therefore, it is necessary to further differentiate the nature of the surprise response and temporal accumulation in the offset positivity.

It is worth noting that the interpretation of the offset P3b as reflecting temporal evidence is specific to the context of a prospective timing task, where the decision threshold is predetermined and the critical event is immediately monitored upon the stimulus onset. In many everyday timing tasks, we may not know what our decision threshold is or we may not be constantly monitoring every event. For instance, we may not realize we have overcooked noodles or missed an appointment until after the fact, when our attention returns to the event and we are surprised at having missed the decision threshold. This type of retrospective timing differs from the prospective timing process (Shi et al., 2022). In prospective timing tasks, judgements of time involve active monitoring of the passage of time, which could be predicted by the classical pacemaker-accumulator model or the drift-diffusion process (Balcı & Simen, 2014). The drift-diffusion model, for example, proposed that the interval timing process is governed by two competing Poisson processes (one excitatory and one inhibitory). The ratio of these processes determines the rate at which an accumulator increases, and this accumulator is continuously monitored if it reaches a predetermined threshold. However, when we judge the duration of a past event retrospectively, we rely on relationships between past events stored in our episodic memory. It is unlikely that each past event would trigger a diffusion process, as this would be inefficient in terms of cognitive resources. Shi et al. (2022) proposed that retrospective timing may rely on oscillatory patterns with computational accessibility to timestamp individual past events, rather than using individual accumulation processes. Estimating the duration of a past event involves reading out oscillatory patterns that are associated with the onset and offset of the event. Therefore, prospective and retrospective time differ in terms of both the timing process and the source of surprise. Results from a prospective temporal bisection task may not be applicable to retrospective timing. Future studies that compare prospective and retrospective timing may help to determine whether the offset P3b activation is a result of the cumulative process or a signature of surprise, or both.

In sum, Ofir and Landau (2022) established a clear connection between neural activity related to decision-making and behavioral performance in a temporal bisection task, showing that the stimulus offset activation is related to the relative distance to the decision boundary in the accumulation-to-bound process. The temporal decision is context dependent, and so is the conclusion drawn from it—we should be cautious on generalizing the relation between the offset activity and the temporal accumulation. Future research that builds on the findings of Ofir and Landau (2022) by using different timing paradigms, such as retrospective versus prospective judgments, would differentiate the nature of the surprise response and temporal accumulation, and further enrich our understanding of neural mechanisms underlying the timing process.
